# Using functional traits and phylogeny to understand local extinction risk in dragonflies and damselflies (Odonata)

**DOI:** 10.1002/ece3.8648

**Published:** 2022-03-06

**Authors:** Jukka Suhonen, Jaakko J. Ilvonen, Esa Korkeamäki, Christina Nokkala, Jukka Salmela

**Affiliations:** ^1^ Department of Biology University of Turku Turku Finland; ^2^ Finnish Environment Institute SYKE Helsinki Finland; ^3^ Water and Environment Association of the River Kymi Kouvola Finland; ^4^ Regional Museum of Lapland Arktikum Finland

**Keywords:** Finland, insect, life history, specialization, species traits

## Abstract

Understanding the risk of local extinction of a species is vital in conservation biology, especially now when anthropogenic disturbances and global warming are severely changing natural habitats. Local extinction risk depends on species traits, such as its geographical range size, fresh body mass, dispersal ability, length of flying period, life history variation, and how specialized it is regarding its breeding habitat. We used a phylogenetic approach because closely related species are not independent observations in the statistical tests. Our field data contained the local extinction risk of 31 odonate (dragonflies and damselflies) species from Central Finland. Species relatedness (i.e., phylogenetic signal) did not affect local extinction risk, length of flying period, nor the geographical range size of a species. However, we found that closely related species were similar in hind wing length, length of larval period, and habitat of larvae. Both phylogenetically corrected (PGLS) and uncorrected (GLM) analysis indicated that the geographical range size of species was negatively related to local extinction risk. Contrary to expectations, habitat specialist species did not have higher local extinction rates than habitat generalist species nor was it affected by the relatedness of species. As predicted, species’ long larval period increased, and long wings decreased the local extinction risk when evolutionary relatedness was controlled. Our results suggest that a relatively narrow geographical range size is an accurate estimate for a local extinction risk of an odonate species, but the species with long life history and large habitat niche width of adults increased local extinction risk. Because the results were so similar between PGLS and GLM methods, it seems that using a phylogenetic approach does not improve predicting local extinctions.

## Graphical abstract

Our results from phylogenetic generalized least squares (PGLS) models indicated that the geographical range size of species was negatively related to local extinction risk of odonates. In contrast, habitat specialist species had lower local extinction rates than habitat generalist species, and it was independent from the relatedness of species. As predicted, species’ long larval period increased the local extinction risk when evolutionary relatedness was controlled.

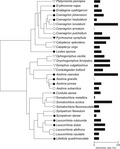

## 1 INTRODUCTION

Anthropogenic disturbances and global warming are rapidly destroying and changing habitats all over the world. Large natural environments are suddenly fragmenting into smaller habitats that are continuously being polluted while being threatened by even more human‐caused changes. Due to these environmental changes, terrestrial insect abundance has declined rapidly (Kwak et al., 2020; van Klink et al., 2020). In addition, freshwater habitats, such as lakes and rivers, have become one of the most degraded habitat types on the planet (Dudgeon et al., 2006). Habitat loss has led to species abundance and biomass loss that has finally led to species extinctions (Cardoso et al., 2020; Seibold et al., 2019; van Klink et al., 2020; Wagner et al., 2021). This can be seen most clearly in the large number of species extinctions in freshwater habitats (Ricciardi & Rasmussen, 1999) resulting in a situation, where freshwater species extinctions are more common than terrestrial species extinctions (Abell, 2002; Ricciardi & Rasmussen, 1999; Richter et al., 1997, but see van Klink et al., 2020).

Understanding the risk of a local extinction, that is, the destruction of a single population of a species, is one of the most important aspects of conservation biology because given finite resources, it helps in prioritizing which species to protect and which habitats to conserve. Therefore, knowing what traits affect the local extinction risk of a species is vitally important when planning cost‐efficient conservation measures (Rocha‐Ortega et al., 2020).

However, local extinction risk studies of species are rare particularly for insects. As a result, relatively few comparisons exist between old faunistic studies and current resurveys (Ball‐Damerow et al., 2014; Korkeamäki & Suhonen, 2002; Suhonen et al., 2010, 2014).

There are three nonmutually exclusive types of variables that may account for variation in extinction risk among species: (i) life history and physiological factors, (ii) ecological factors such as intra‐ and interspecific interactions, and (iii) environmental factors (Chichorro et al., 2019; Rocha‐Ortega et al., 2020). So far previous studies that have used a wide range of ecological factors have found that geographical range size (henceforth GRS) is the best overall predictor of an extinction risk, niche breadth being the second best predictor (Chichorro et al., 2019; Kotiaho et al., 2005; van Swaay, 1990). Unfortunately, most of these studies have no real data on the extinction risk of local populations but are based on extrinsic factors, namely population loss and decline of geographical range size (Rocha‐Ortega et al., 2021). In addition, most of these studies have used a simple comparison between threatened and nonthreatened species (Chichorro et al., 2019) due to fact that more detailed knowledge of species traits was not available in most of the insect orders (Mattila et al., 2006). The successful identification of traits that are linked to an insect species’ extinction risk can potentially be applied to other species groups and may be used to develop accurate and cost‐efficient species‐specific conservation strategies.

With insects, extinction risk seems to be affected by several traits, such as GRS, habitat niche breadth, length of flying period, life history, dispersal ability, and body size (Chichorro et al., 2019; Grewe et al., 2013; Hof et al., 2006; Korkeamäki & Suhonen, 2002; Kotiaho et al., 2005; McCauley et al., 2014; Outomuro & Johansson, 2019; Rocha‐Ortega et al., 2020; Rundle et al., 2007; Suhonen et al., 2010, 2014; Swaegers et al., 2014; van Swaay, 1990). Unfortunately, there is very limited knowledge on the GRS and its temporal changes for most insect and invertebrate species (Grewe et al., 2013; Hof et al., 2011; Pöyry et al., 2009). This means that future studies should incorporate alternative traits with different combinations to better evaluate the extinction risk of invertebrate species.

Dragonflies and damselflies (Odonata) are particularly good candidates for comparing the local extinction risk with species traits (Ball‐Damerow et al., 2014; Korkeamäki & Suhonen, 2002; Suhonen et al., 2010, 2014). Previous odonate studies have found that population numbers decline over time, if a species is a habitat specialist, has a narrow GRS, large body size, narrow thermal limits, and an overwintering diapause (Ball‐Damerow et al., 2014; Korkeamäki & Suhonen, 2002; Rocha‐Ortega et al., 2020; Suarez‐Tovar et al., 2019; Suhonen et al., 2010, 2014).

To further examine this topic, we analyzed the local extinction risk of dragonflies and damselflies and created a large dataset of six different species traits of which three were not included in our previous studies (Korkeamäki & Suhonen, 2002; Suhonen et al., 2010, 2014). We further developed our approach by controlling the phylogeny of odonate species. We used a phylogenetic approach for two reasons. First, the lack of independence between the study species can affect a species’ morphological and ecological traits and affect our results. Second, we wanted to know whether a phylogenetic approach can improve predicting the risk of local extinctions.

In this study, we answer the following questions: (i) how different species traits influence the local extinction risk of odonate species? We chose six different ecological, life history, and morphological traits: primary larval habitat (Habitat), adult habitat (hereafter Niche), geographical range size (GRS), hind wing length (Wing), length of adult flying period (FTime), and length of larval period (Larvae) to evaluate how they affect the local extinction risk (henceforth LER) of each study species (Table 1). Detailed predictions of each trait are listed in Table 1. (ii) Are closely related odonate species more similar in these biological and ecological traits than species drawn at random? i.e., is phylogeny a factor that has to be taken into account in extinction assessments? and (iii) can these traits and species relatedness be used to predict the future local extinction risk of insect species? Based on our previous results (Korkeamäki & Suhonen, 2002; Suhonen et al., 2010, 2014), we expected that species with larger GRS have a lower local extinction probability than species with a smaller GRS. We also expected that habitat generalist species have a lower local extinction risk than habitat specialist species. We also expected that closely related species are more similar in their biological and ecological traits, suggesting that phylogenetic approaches are needed in future extinction analyses.

**TABLE 1:** Predicted direction of five traits in relation to local extinction risk (LER)

Trait	Prediction	References
GRS	LER decreases with increasing GRS due to higher colonization rate	Korkeamäki and Suhonen (2002), Mattila et al. (2006), Suhonen et al. (2014), Chichorro et al. (2019)
Larvae	Longer generation time increases LER due to higher predation risk during larvae period	Jeppsson and Forslund (2014)
Wing	High dispersal ability decreases LER due to higher colonization rate	Kotiaho et al. (2005)
FTime	Longer flying time decreases LER due to longer colonization period	Kotiaho et al. (2005), Mattila et al. (2006), Mattila et al. (2008), Jeppsson and Forslund (2014)
Niche	Adult niche large or narrow. Species which had large adult niche have lower LER than specialist ones due to higher possibility to find suitable habitat for breeding	Chichorro et al. (2019)
Habitat	Main larvae habitat is standing or running water. Species which larvae mainly occurred in the standing water have lower LER than running water ones due to higher predictability and lower disturbances	Korkeamäki and Suhonen (2002), Rocha‐Ortega et al. (2020)

Note

The traits are geographical range size (GRS), duration of the larval period (Larvae), hind wing length (Wing), length of flying period (FTime), Niche indicates whether a species is a habitat generalist (G), or a habitat specialist (S).

## 2 METHODS

### 2.1 Local extinction risk

Local extinction (LER) risk was assessed by comparing the existence/absence of local populations of 19 dragonfly and 12 damselfly species (Odonata) in Central Finland. First population surveys of Finnish Odonata were mostly conducted from 1930s to 1950s, but were extended to 1975, and resurveyed again between 1995 and 2003 (Korkeamäki & Suhonen, 2002; Suhonen et al., 2010, 2014). Initial surveys found 548 populations, of which 301 populations were located in 23 different ponds and lakes (standing water) and 232 population in 34 small creeks and brooks (running waters), and 15 populations in three bogs (Table 1). All studied waterbodies were located within 150 km of each other (see Figure 1 in Suhonen et al., 2010). All studied waterbodies were permanent. It is possible that local extinctions are spatially autocorrelated, particularly if the local populations are spatially aggregated (e.g., Kallimanis et al., 2005) and the co‐occurring species have very similar traits. It seems, however, that this explanation is unlikely in our case because the local extinctions occurred most often in low‐quality habitat patches with species that mostly had a wide niche bread (see more details in Suhonen et al., 2010), and there were very few species extinction in the same waterbodies.

**FIGURE 1:**
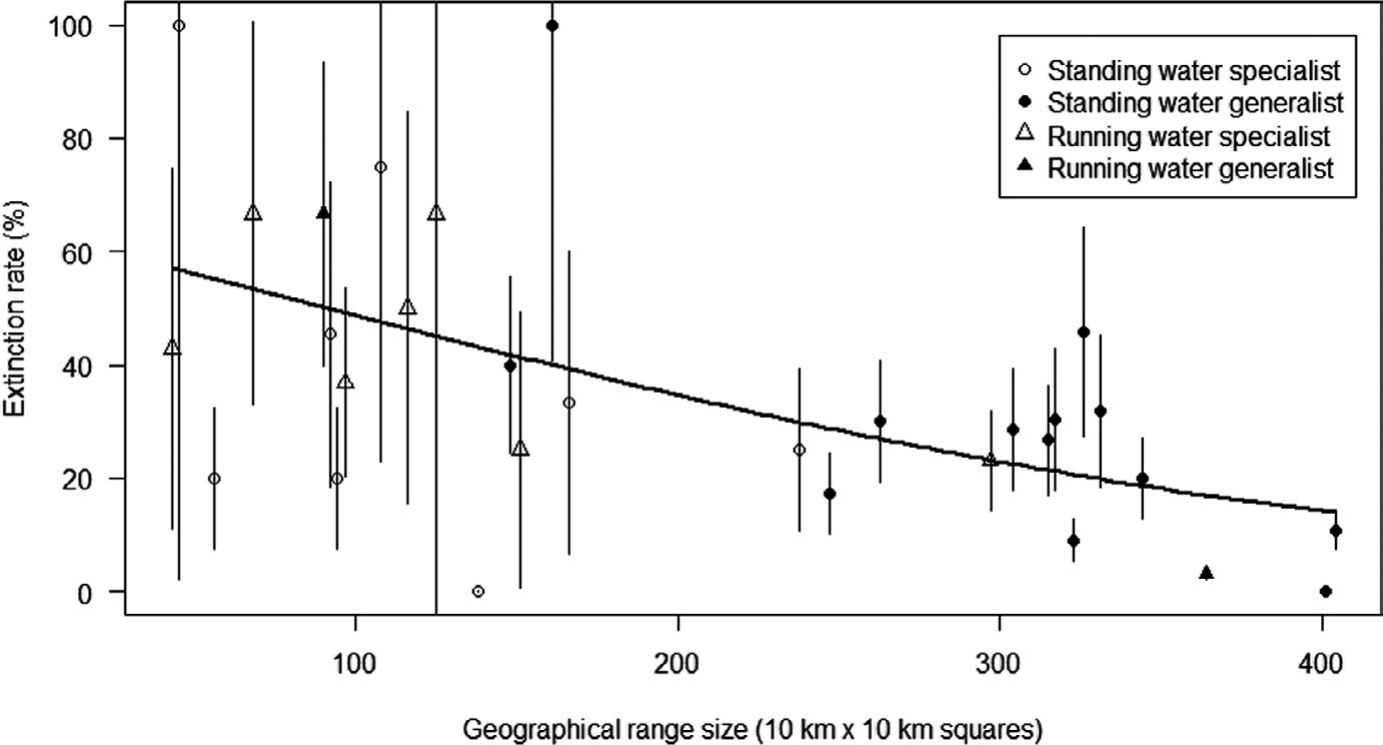
The relationship between the probability of a population's local extinction rate (%) and their 95% confidence intervals for the 31 odonate species and their geographical range size (number of 10 km × 10 km squares) in Finland. The continuous trendline indicates most fitted value in the logistic regression. Dots denote that primary larval main habitat is standing water, and triangles denote that primary larval habitat is running water. White symbol denotes adult niche specialist, and black symbol denotes adult niche generalist

### 2.2 Species traits

Although old odonate surveys relied mainly on adult observations, we also included the presence of larvae to increase the accuracy of habitat viability (see e.g., Bried et al., 2015, Patten et al., 2015). Primary larval habitat (Habitat for short) was divided into two: standing water (22 species) and running water (9 species) habitats (Table 2). These data were based on Valle's works (1952) and our previous publications (Korkeamäki & Suhonen, 2002; Suhonen et al., 2010, 2014) from the same area in Central Finland.

**TABLE 2:** In total, 31 odonate species used in this study from Central Finland and their trait values

Species	Habitat	Niche	Suborder	GRS	Larvae	Wing	FTime	Pop	Ext
*Aeshna caerulea*	S	W	A	161	4.5	38.5	82	11	11
*Aeshna grandis*	S	W	A	401	2.5	46.0	113	26	0
*Aeshna juncea*	S	W	A	263	5.0	45.5	127	30	9
*Aeshna subarctica*	S	N	A	94	4.0	43.5	104	10	2
*Calopteryx splendens*	R	N	Z	116	2.5	31.5	99	8	4
*Calopteryx virgo*	R	N	Z	297	2.5	32.0	96	26	6
*Coenagrion armatum*	S	N	Z	138	3.0	19.0	73	7	0
*Coenagrion hastulatum*	S	W	Z	404	2.0	19.5	99	37	4
*Coenagrion johanssoni*	S	W	Z	148	2.0	17.5	84	25	10
*Coenagrion lunulatum*	S	N	Z	45	1.5	20.0	70	4	4
*Coenagrion pulchellum*	S	N	Z	166	1.5	19.5	87	6	2
*Cordulegaster boltoni*	R	N	A	97	4.5	45.5	46	19	7
*Cordulia aenea*	S	W	A	315	2.5	33.0	93	30	8
*Enallagma cyathigerum*	S	W	Z	331	2.0	20.5	121	22	7
*Erythromma najas*	S	W	Z	247	1.0	22.5	91	23	4
*Gomphus vulgatissimus*	R	N	A	125	3.0	31.0	82	3	2
*Lestes sponsa*	S	W	Z	317	1.0	21.5	87	23	7
*Leucorrhinia albifrons*	S	N	A	92	3.0	29.5	86	11	5
*Leucorrhinia caudalis*	S	N	A	94	3.0	31.0	68	10	2
*Leucorrhinia dubia*	S	W	A	326	3.0	25.5	98	24	11
*Leucorrhinia rubicunda*	S	W	A	304	2.5	29.0	92	28	8
*Libellula quadrimaculata*	S	W	A	344	2.0	35.0	106	30	6
*Onychogomphus forcipatus*	R	N	A	68	4.0	30.5	73	15	10
*Ophiogomphus cecilia*	R	N	A	43	3.5	33.0	48	7	3
*Platycnemis pennipes*	R	N	Z	151	1.0	21.5	96	4	1
*Pyrrhosoma nymphula*	R	W	Z	90	2.0	22.0	68	24	17
*Somatochlora arctica*	S	N	A	108	3.0	33.5	85	8	6
*Somatochlora flavomaculata*	S	N	A	56	3.0	36.5	85	10	2
*Somatochlora metallica*	R	W	A	364	3.0	36.0	102	33	1
*Sympetrum danae*	S	W	A	323	1.0	24.0	99	22	2
*Sympetrum flaveolum*	S	N	A	238	1.0	26.0	99	12	3

Note

The “Primary larval habitat” indicates standing water (S) or running water (R), “Niche” indicates whether adults have a wide niche (W) (flying frequently in both standing and running waters) or a narrow niche (N) (flying primarily in a single habitat type), “Suborder” indicates whether a species is a dragonfly (A) or a damselfly (Z), “GRS” means geographical range size as the number of 10 km × 10 km squares in Finland, “Larvae” means duration of the larval period in years, “Wing” indicates the average hind wing length (mm), “FTime” means the length of flying period during summer in days, “Pop” indicates the number of original local populations, and “Ext” mean the number of populations vanished between 1930 and 1975 and 1995 and 2003.

Adult habitat bread (Niche), that is, the presence of flying adults, was divided into narrow (primarily a single habitat type: standing or running water) and wide (flying frequently in both habitat types) niche breadth. (Table 2). These data were based on our previous publications (Suhonen et al., 2010, 2014).

The geographical range size was measured according to the previously published distribution maps for each of the 31 studied species in Finland. Although the studied water bodies were situated in Central Finland, we found it important to get an estimate of the GRS due to its importance in predicting extinction probabilities. Because there were no reliable maps specific to both study periods in our study area, we used the distribution maps provided by Valtonen (1980). They were considered the most accurate because they are based on an extensive atlas on damselfly and dragonfly distribution in Finland from the late 1880s up to 1979, and it matches the time frame between old and resurvey periods. Moreover, species composition has not changed drastically between the original and resurvey periods because the species GRS data by Valtonen (1980) correlated well with the current (2021) and continuously updated GRS data (*r* = 0.94, *n* = 31, *p* < .001) (www.laji.fi, Finnish Biodiversity Info Facility). The GRS of the species is presented as a number of occupied 10 × 10 km (standardized coordinate system in Finland) squares in each species distribution map (Valtonen, 1980). Each occupied square was considered a separate unit, and they were tallied for each species.

The mean hind wing length was calculated for each study species from the minimum and maximum values presented in the textbook “Dragonflies of Finland” (Karjalainen, 2010). Previous comparative studies have found that wing length and its morphological variations between species is a proxy for dispersal ability of odonates (Grewe et al., 2013; Hof et al., 2006; McCauley et al., 2014; Outomuro & Johansson, 2019; Rundle et al., 2007; Swaegers et al., 2014) and a species’ body size (Aromaa et al., 2019).

The length of adult flying period (number of days) was estimated from figures published in the textbook “Dragonflies of Finland” (Karjalainen, 2010), and it means the time span when adult odonates are actively flying during the summer in Finland.

The length of larval period, that is, the number of years an odonate spends in the water before emergence, was based on a published dataset of which we used the most northern ones (Corbet et al., 2006). If the species had two different estimations from northern populations for the length of larval period, we calculated a mean value and used it in the statistical tests.

### 2.3 Statistical analyses

Because the elapsed time between sampling and resampling may affect LER, we used a binary logistic regression to evaluate whether it affects a species’ LER. In this test, the elapsed time (years) between the first record of a local population and the resurvey of the population was used as continuous covariate, and each local population was independent variable, extinct (0) or survived (1).

Because it was not possible to use phylogenetic binary logistic regression analysis to estimate the effect of species traits on LER, we used an alternative Poisson distribution approach also in generalized linear models with type III errors. In this statistical model, the link function was log, the probability distribution was Poisson, the number of vanished populations was the dependent variable, covariates were the number of old populations, GRS, hind wing length, length of flying period and larval period, and the adult habitat bread (narrow/wide) and primary larvae habitat (standing/running water) were used as factors. An alternative suitable solution for data analysis is to conduct a logistic regression in which the dependent variable is the proportion of populations that went extinct. We used this approach to estimate the extinction probability in the figure. We compared differences between the adult habitat bread (narrow/wide) and primary larvae habitat (standing/running water) in species’ GRS and their extinction rates (%) with a *t*‐test. For each species, the extinction rate was calculated using the following formula:

1. Extinction rate of a species (%) = 100*(# vanished populations/# surveyed populations).

The phylogenetic tree used in our study was pruned from a larger tree created by Waller and Svensson (2017). Unfortunately, *Aeshna caerulea* was absent from this tree and had to be replaced by its close relative (*Aeshna cyanea*) that was present in the tree.

For continuous species traits (LER, GRS, hind wing length, length of flying period, and length of larvae period), we used Pagel's lambda (*λ*) (Freckleton et al., 2002; Pagel, 1999) to measure the phylogenetic signal. A *λ* ‐value near 0 indicates that trait values vary randomly across a phylogeny (i.e., absence of phylogenetic signal), while a *λ*‐value near 1 indicates Brownian motion of evolution (i.e., the presence of phylogenetic signal) (Freckleton et al., 2002). This metric of phylogenetic signal performs well in statistical tests for evolutionary trait conservatism (Muenkemueller et al., 2012). We estimated index values and tested for deviations from 0 in R, using the “phytools” package (Revell, 2012) for Pagel's *λ*.

To measure phylogenetic conservatism in the binary variables, adult habitat bread, (narrow/wide) and primary larvae habitat (standing/running water), we used the *D*‐statistic (Fritz & Purvis, 2010). Using this method, a *D*‐value close to 0 indicates a phylogenetically clustered pattern expected under a Brownian threshold model, whereas a value close to 1 indicates a phylogenetically random pattern (Fritz & Purvis, 2010). We evaluated for deviations from 0 and 1 in R, using the “caper” package (Orme, 2018) for the *D*‐statistic.

Due to the shared ancestry, the study species could not be considered independent data points, and therefore phylogenetic least‐square (PGLS) analyses was used to assess the relationship between LER and species traits. However, for our results to be comparable with previous studies, we also used nonphylogenetic analyses, that is, generalized linear models (GLMs) that treat each species as an independent data point (Table 1).

Because it was not possible to use phylogenetic binary logistic regression analysis to estimate the effect of species traits on LER, we used an alternative Poisson distribution approach. Using a phylogenetic Poisson regression model (phyloglm function of the “phylolm” v.2.6 package (Ho & Ane, 2014)) with the "poisson_GEE" method (Ives & Garland, 2010), we first evaluated whether the number of original study populations affected the number of extinct populations. As expected, there was a strong effect (GLMs, χ^2^ = 8.98, df = 1, *p* = .003), and therefore we used this model as our baseline model (BM) to which we added other traits such as GRS, hind wing length, breeding habitat preference status, length of flying period, and larval period.

Phylogenetic analyses and tree manipulations were performed in the R programming environment (version 4.0.2) (RCore, 2018) using RStudio (version 1.3.1073) and the "ape" (Paradis et al., 2004), "caper" (Orme, 2018), "geiger" (Harmon et al., 2008), "ggplot2" (Wickham, 2016), and "phytools" (Revell, 2012) packages.

We used AICc (Akaike Information Criterion for small sample sizes) to compare the basic model (BM) with the number of old populations to alternative models to find the model that best explained the LER of each species. This approach can detect more fitted models than the baseline model if ΔAICc (= AICc (BM) – AICc (alternative)) values are lower than 4 strongly selected models and with 4–7 being where models lack strong support but still may be worth considering (Anderson et al., 2000; Burnham et al., 2011; Burnham & Anderson, 2000). All analogous nonphylogenetic Poisson regression analyses were performed using IBM SPSS Statistics for Windows version 26.

## 3 RESULTS

In total, 164 out of the 548 original populations went extinct during the study period. On average, 5.3 local populations vanished from each odonate species (SD = 3.9; *n* = 31 species, range 0–17; Table 1), meaning that each species lost one third of their populations (mean = 35.9%, SD = 26.0, range 0–100%; Table 1; Figure 1). Sixty‐six out of 209 damselfly (suborder Zygoptera) populations and 98 out of 339 dragonfly (suborder Anisoptera) populations went extinct during the study period. On average, 5.5 (SD = 4.6, range 0–17) damselfly and 5.2 (SD = 3.6, range 0–11) dragonfly populations vanished. Elapsed time (years) between the original survey and resurvey did not affect the local extinction risk of populations (Logistic regression, Wald = 1.04, df = 1, *p* = .309), so we omitted this variable from further statistical analyses.

Based on adult surveys, there were 14 species with wide niche breadth and 17 species with narrow niche breadth that were further divided into running water (*n* = 7) and standing water (*n* = 10) species. Species with wide adult niche breadth had larger GRS (mean = 287, SD = 95, *n* = 14) than species with narrow adult niche breadth (mean = 132, SD = 83, *n* = 14) (*t*‐test, *t* = 4.82, df = 29, *p* < .001; Figure 1). However, extinction rate did not differ between wide niche (mean = 29.9%, SD = 26.9, *n* = 14) and narrow niche species (mean = 40.9, SD = 25.0, *n* = 17). (*t*‐test, *t* = −1.18, df = 29, *p* = .247; Figure 1).

Standing water species had a slightly larger GRS (223.4, SD = 115.6, *n* = 22) than running water species (150.1, SD = 108.3, *n* = 9) (*t* = 1.63, df = 29, *p* = .114). Running water species had slightly higher local extinction rates (42.8, SD = 23.2, *n* = 9) than standing water species (33.2, SD = 27.1, *n* = 22), but the difference was not statistically significant (*t* = −0.93, df = 29, *p* = .359).

**FIGURE 2:**
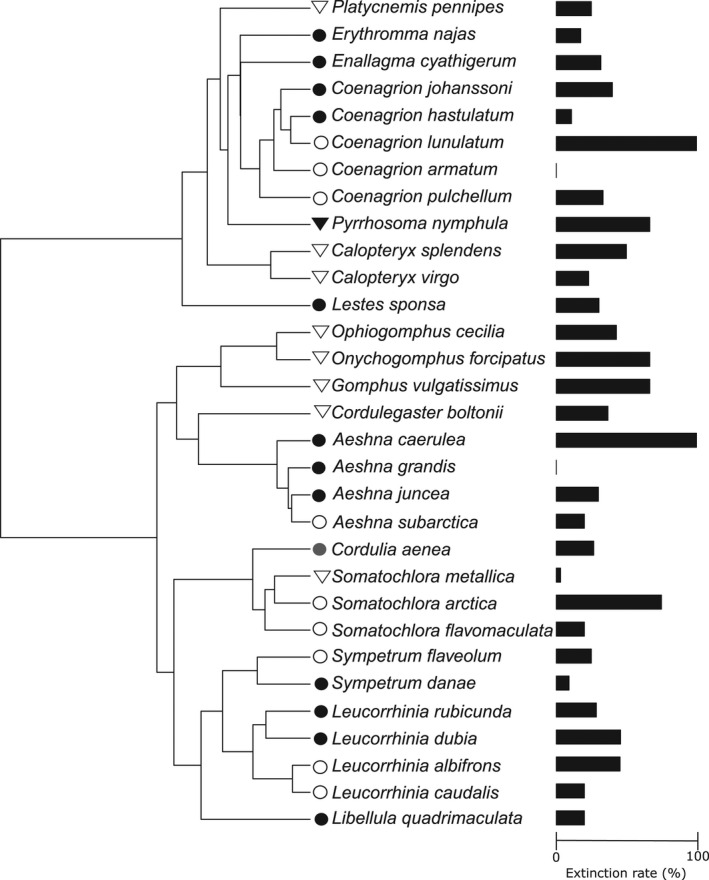
Phylogeny of the odonate species used in this study and the local extinction rate (%). Bars represent the local extinction probability (%) of each species. Dots denote that primary larval habitat is standing water, and triangles denote that primary larval habitat is running water. White symbol denotes adult niche specialist, and black symbol denotes adult niche generalist. Phylogeny is based on the odonate tree by Waller & Svensson, 2017 (see also Material and Methods section for further details)

Phylogenetic signals were present in hind wing length (*λ* = 1.05, *p* < .001) and length of larval period (*λ* = 0.81, *p* < .001), but were absent in LER (*λ* ≈ 0.00, *p* ≈ 1.00; Figure 2), GRS (*λ* ≈ 0.00, *p* ≈ 1.00), and length of flying period (*λ* ≈ 0.00, *p* ≈ 1.00). The adult habitat bread was random with respect to odonate phylogeny (*D* = 1.10, *p*(0) = .006, *p*(1) = .6197). However, primary larvae habitat had phylogenetic signal (*D* = −0.08, *p*(0) = .5668, *p*(1) = .0017) indicating that closely related species had similar larval habitat.

Results from six separate traits (Table 3) fitted very well with our predictions (Table 1). Predictably, the number of resurvey populations increased LER, and for that reason, it was kept in all the models (Table 3). The most important individual trait was GRS, which had the lowest AICc value. All other traits fitted less well with the data (ΔAICc ≥ 13.48; Table 3) although all separate trait models were statistically significant (Table 3). Length of flying time and wing length decreased LER, and length of larvae period increased LER. The only exception with our prediction was that habitat specialist species had similar LER compared with habitat generalist species (Table 3).

**TABLE 3:** General linear model (all species as independent observations, *n* = 31) results in which the dependent variable was the number of extinct populations of each odonate species

General linear model					
Variable	*χ* ^2^	df	AICc	ΔAICc	Slope
Pop	11.07	1	181.52		
Pop + GRS	31.84	2	163.21	0.00	−**0.005**
Pop + FTime	18.36	2	176.69	13.48	−**0.013**
Pop + Larvae	15.71	2	179.34	16.13	**0.154**
Pop + Habitat (Standing)	12.17	2	182.89	19.68	−0.180
Pop + Niche (Narrow)	11.85	2	183.20	19.99	−0.215
Pop + Wing	11.68	2	183.87	20.66	−0.007

Note

Explanatory variables were the number of populations (Pop), geographical range size (number of 10 km x 10 km squares in Finland) (GRS), hind wing length (mm) (Wing), primary larval habitat (running water or standing water) (Habitat), adult niche breadth (narrow, primarily one type of habitat) or wide (standing and running water) (Niche), length of larval periods in years (Larvae), and length of flying period (days) (FTime). The model with the number of populations (Pop) was analyzed alone. All other models included the number of populations (Pop), and other explanatory variables were analyzed separately. All presented models were statistically significant at level, *p* < .001. The ΔAICc values indicate a better fit with the data than our baseline model, where Pop was the explanatory variable. The most fitted model with the lowest AICc value is presented in bold. The statistically significant slope value (*p*< .05) is in bold.

Both the Poisson regression (Table 4) and the phylogenetic Poisson regression (Table 5) results were very similar. In the full model that included all trait variables, AICc value was 160.2. Most of the studied species’ traits influenced LER (Tables 4 and 5). As expected, the number of locally vanished populations increased with increasing number of studied populations (Tables 4 and 5). The GRS seems to be the most important trait affecting a species’ LER: the local extinction risk of a population decreases significantly with increasing GRS in both uncorrected and phylogenetically corrected analyses (Tables 4 and 5; Figures 1 and 2), respectively. The long larval period on the other hand increased LER in both analyses (Table 4 and 5). Wing length decreased LER (*p* < .05; Table 4) and was achieved only after correcting for phylogeny (Table 5). Interestingly, adult habitat specialist species had a similar LER compared with adult habitat generalist species (Tables 4 and 5; Figure 2). The length of the flying period did not affect local extinction risk in either uncorrected or phylogenetically corrected analyses (Tables 4 and 5).

**TABLE 4:** Results from full generalized linear models (all species as independent observations)

Parameter	Estimate	SE	95% Confidence Interval		Hypothesis Test		
			Lower	Upper	Wald	df	*p*
Intercept	2.439	0.562	1.337	3.541	18.825	1	**<.001**
Pop	0.032	0.017	−0.001	0.065	3.693	1	.055
Larvae	0.298	0.125	0.052	0.544	5.658	1	.**017**
GRS	−0.004	0.001	−0.006	−0.001	6.880	1	.**009**
Wing	−0.030	0.016	−0.061	0.002	3.470	1	.063
FTime	−0.002	0.006	−0.014	0.011	0.055	1	.814
Niche (Wide)	0.838	0.280	1.388	0.289	8.948	1	.**003**
Habitat (Standing)	−0.097	0.213	−0.515	0.321	0.209	1	.648

Note

Dependent variable was the number of local populations vanished. Explanatory variables were the number of old populations (Pop), geographical range size (number of 10 km × 10 km squares in Finland) (GRS), hind wing length (mm) (Wing), length of flying period during summer in days (FTime), mean length of larval periods in years (Larvae), and primary larval habitat (Habitat) (running water or standing water). Adult niche, narrow (primarily one habitat type) or wide (both running and standing waters) (Niche). The statistically significant value (*p* < .05) is in the bold.

**TABLE 5:** Results from the full phylogenetic general linear model with phylolm function and "Poisson_GEE" method (Ives & Garland, 2010)

Variable	Estimate	SE	z‐value	*p*
Intercept	1.269	0.493	2.57	.**010**
Pop	0.030	0.010	2.95	.**003**
Larvae	0.394	0.092	4.26	**<.001**
GRS	−0.005	<0.001	−5.17	**<.001**
Wing	−0.034	0.014	−2.38	.**017**
FTime	0.002	0.004	0.56	.576
Niche (Wide)	0.825	0.193	4.27	**<.001**
Habitat (Standing)	0.264	0.173	1.53	.127

Note

Dependent variable was the number of vanished local populations. Explanatory variables of each species were the number of populations studied (Pop), length of larval period in years (Larvae), geographical range size (number of 10 km × 10 km squares in Finland) (GRS), hind wing length (mm) (Wing), length of flying period in days) (FTime), primary larval habitat (Habitat, standing water (*w*) or running water (*r*)), adult niche breadth (Niche, wide (*w*) or narrow (*n*)). The statistically significant value (*p*< .05) is in the bold.

## 4 DISCUSSION

We found that several species traits influenced the local extinction risk of a species. Like previous findings (Korkeamäki & Suhonen, 2002; Suhonen et al., 2014), larger geographical range size of a species decreased the chance of a local extinction of a population. Longer larval period increased local extinction risk, whereas wing length decreased and the length of the flying period had no effect on it. Opposite to our prediction, species with narrow adult niche had a lower extinction risk compared to species with large niche. Finally, phylogenetic signals were present in morphological and life history traits, but not in ecological traits except larvae main habitat.

### 4.1 Geographical range size

Based on our data, smaller geographical range size of a species strongly increases the LER. Our result may be explained by the fact that abundance and GRS tend to be correlated, abundant species being more often widely distributed (Blackburn et al., 1998; Gaston, 2003). So, a larger GRS helps to buffer against LER by increasing ecological opportunities whereas a smaller GRS limits that opportunity. Moreover, in some cases, highly mobile and widespread odonate species have become increasingly common in areas affected by anthropogenic disturbance, such as eutrophication (Suhling et al., 2006). However, most often high anthropogenetic disturbances destroy freshwater habitats that leads to high local extinction rates. These results give support to previous findings where a narrow GRS indicated a high extinction risk for a species (Chichorro et al., 2019; Mattila et al., 2006) and our previous results from odonates (Korkeamäki & Suhonen, 2002; Suhonen et al., 2014, but see Rocha‐Ortega et al., 2021).

### 4.2 Length of larval period

According to our findings, a longer larval period increased the local extinction risk. Our results on the effect of longer larval duration, that is, a longer generation cycle, is similar to a study of Swedish longhorn beetles (Cerambycidae) that found an increased extinction risk with a longer generation time (Jeppsson & Forslund, 2014). The long larval period is also closely linked with large body size in odonates (Corbet et al., 2006). Although larger body size generally increases species dispersal distance, it seems that in our study system it has not been able to compensate for the potential negative aspect of other body size‐related factors. It is possible that larger species have a lower local density compared with smaller species (e.g., Corbet, 1999) resulting in a smaller local population size. Longer aquatic larval duration is likely to increase predation risk of top aquatic predators (e.g., fishes) and decrease the size of the local odonate population. It has been shown in numerous studies that low population size increases LER (e.g., Hanski, 1999). Moreover, low population size probably decreases species’ dispersal ability by limiting the number of individuals that colonize new habitats thus affecting their GRS. Species’ body size is one of the most used species traits in extinction risk evaluations, and our results support previous findings that large species have a higher extinction risk than smaller species (Chichorro et al., 2019; Rocha‐Ortega et al., 2020; Suarez‐Tovar et al., 2019). However, this relationship has not been found in Finnish butterflies when comparing threatened and nonthreatened species and their body sizes (Kotiaho et al., 2005), highlighting the need for further studies on the role of body mass size on insect extinction risk (Chichorro et al., 2019).

### 4.3 Main habitat of larvae

In this study, we did not observe differences in local extinction rates between species, which larvae mainly occurred in the standing water or running water habitat. This is surprise a result because it was estimated that running water species had higher extinction risk in the northern America (Rocha‐Ortega et al., 2020). Moreover, our results did not support the idea that standing water species have large GRS than running water species (Hof et al., 2006).

### 4.4 Dispersal ability

The phylogenetically corrected analysis, and the noncorrected to a certain degree, showed that odonate species with longer wings have a lower LER. Overall, habitat loss through anthropogenic freshwater use is a major cause of local extinctions of odonates in modern landscapes (Ball‐Damerow et al., 2014; Korkeamäki & Suhonen, 2002; Suhonen et al., 2014). Reduced area and connectivity of natural habitats limit the colonization of empty and suitable habitat patches especially with species that have a low dispersal ability (Hanski, 1999; Hanski & Ovaskainen, 2000). Odonate species dispersal behavior is also closely linked to larger GRS (Grewe et al., 2013; McCauley et al., 2014; Outomuro & Johansson, 2019; Rundle et al., 2007; Swaegers et al., 2014). Moreover, a comparative odonate species studies found that species with a high extinction risk ratio in relation to colonization ratio had relatively small geographical GRS and *vice versa* (McCauley et al., 2014). Our results also support a previous butterfly study that found that the poor dispersal ability of a species increased its extinction risk (Kotiaho et al., 2005).

### 4.5 Length of flying period

We did not find any evidence that the length of flying period affected the local extinction risk. Our results were opposite than several previously published insect studies done on butterflies (Kotiaho et al., 2005), noctuid moths (Mattila et al., 2006), geometrid moths (Mattila et al., 2008), and longhorn beetles (Jeppsson & Forslund, 2014). It is possible that longer adult lifespan may increase the dispersal period subsequently decreasing local extinction risk. However, both ideas require more investigation in the future.

### 4.6 Adult habitat breadth

Specialists have long been regarded as losers, and generalists as winners in the current extinction crisis (Chichorro et al., 2019). Numerous previous studies have found that a narrow breeding habitat range increases extinction risk (Chichorro et al., 2019; Nylin & Bergstrom, 2009). Our results challenge this generalization. We found that adult habitat specialists’ damselflies and dragonflies did not have a higher local extinction risk than habitat generalist species, and it seems that habitat quality may at least partly explain this difference. It was observed that adult habitat generalists, regardless of whether they were damselflies or dragonflies, occurred in high‐ and low‐quality habitats (Suhonen et al., 2010). Unsurprisingly, the local extinction risk was higher in the latter (Suhonen et al., 2014). If species differ in their use of high‐ and low‐quality habitats that also function as sources and sinks for dispersing individuals, it may affect the general likelihood of an extinction. However, given recent studies in conducting accurate odonate surveys (e.g., Bried et al., 2015; Patten et al., 2015), we recognize that the presence of adult individuals does not necessarily indicate that the site is suitable for larvae and successful life cycle completion. Most of the previous odonate records, which we re‐surveyed, were based on adults. This may partly explain why species with wide niche breadth had higher local extinction rates. Furthermore, this may indicate a decrease in population size in the main breeding habitat. Regardless, adults do engage in habitat selection for reproduction and foraging (Corbet, 1999) and being a habitat generalist may thus increase the likelihood of a species being able to find suitable breeding sites in new locations even if the habitat is a low‐quality sink habitat (Gilroy & Sutherland, 2007; Pulliam, 1988; Watkinson & Sutherland, 1995). Despite these sink habitats not being ideal for a species, they may be supportive for at least a part of a species’ life cycle (Gilroy & Sutherland, 2007; Watkinson & Sutherland, 1995), and they may function as “rescue habitats” if the high‐quality source habitat temporarily declines in quality or disappears (Watkinson & Sutherland, 1995). However, without continuous dispersal of individuals from the source to sink habitat, the low quality, sink population will face local extinction (Pulliam, 1988; Watkinson & Sutherland, 1995). Therefore, it seems prudent that future extinction risk studies and conservation efforts should pay even more attention to the quality of habitats and not only on their numbers. In addition, if a threatened species can use both high‐ and low‐quality habitats, conservation efforts should prioritize these high‐quality source habitats over poor‐quality sink habitats if conservation resources are limited. However, if it is possible, conservation efforts should maintain high‐quality habitats and improve low‐quality habitats to maximize the efficiency of a conservation effort.

### 4.7 Phylogenetic signal

We found that the length of larval period, primary larvae habitat, and hind wing length had phylogenetic signals, corroborating previous results (Aromaa et al., 2019; Ilvonen & Suhonen, 2016; Suarez‐Tovar et al., 2019). These findings indicate that intrinsic factors, such as these morphological and life history traits, are nonrandomly distributed in the phylogenetic tree of odonates (Waller & Svensson, 2017).

The LER, GRS, length of flying period, or main habitat of adult did not have a phylogenetic signal similar to a previous study (McCauley et al., 2014). This indicates that these ecological traits are randomly distributed in the odonate phylogeny (Waller & Svensson, 2017). Interestingly, using phylogenetic methods does not appear to improve evaluations on the local extinction risk of species.

## 5 CONCLUSIONS

It seems that habitat loss through anthropogenic land use may be a larger cause of local extinctions of odonate populations in waterbodies (Ball‐Damerow et al., 2014; Korkeamäki & Suhonen, 2002; Suhonen et al., 2010, 2014). However, based on our results, there are several species traits that can be used to evaluate the local extinction risk of an insect species besides its GRS. Although our results confirm several previously found connections between species traits and their extinction probabilities, the differences between our results and those from previous studies highlight the need for more research, especially on aquatic or semi‐aquatic insects. Understanding how habitat requirements (both adults and larvae) and different species traits affect the local (and subsequently global) extinction risk is vital, both to theoretical ecology and to applied ecology, such as conservation biology.

## CONFLICT OF INTEREST

Authors have no conflicts of interest.

## AUTHOR CONTRIBUTION

**Jukka Suhonen:** Conceptualization (equal); Data curation (equal); Formal analysis (equal); Funding acquisition (equal); Methodology (equal); Project administration (equal); Validation (equal); Visualization (equal); Writing – original draft (equal). **Jaakko Ilvonen:** Conceptualization (equal); Formal analysis (equal); Methodology (equal); Writing – original draft (equal). **Esa Korkeamäki:** Conceptualization (equal); Data curation (equal); Funding acquisition (equal); Investigation (equal); Methodology (equal); Validation (equal); Writing – review & editing (equal). **Christina Nokkala:** Methodology (equal); Resources (equal); Validation (equal); Writing – review & editing (equal). **Jukka Salmela:** Data curation (equal); Funding acquisition (equal); Investigation (equal); Validation (equal); Writing – review & editing (equal).

## Data Availability

All necessary data for this study are published, and the empirical data are in the Table 2. The original data are available in https://doi.org/10.5061/dryad.6q573n610.

## References

[ece38648-bib-0001] Abell, R. (2002). Conservation biology for the biodiversity crisis: a freshwater follow‐up. Conservation Biology, 16(5), 1435–1437. 10.1046/j.1523-1739.2002.01532.x 35701979

[ece38648-bib-0002] Anderson, D. , Burnham, K. , & Thompson, W. (2000). Null hypothesis testing: Problems, prevalence, and an alternative. Journal of Wildlife Management, 64(4), 912–923. 10.2307/3803199

[ece38648-bib-0003] Aromaa, S. , Ilvonen, J. J. , & Suhonen, J. (2019). Body mass and territorial defence strategy affect the territory size of odonate species. Proceedings of the Royal Society B‐Biological Sciences, 286(1917), 20192398. 10.1098/rspb.2019.2398 PMC6939934 31847780

[ece38648-bib-0004] Ball‐Damerow, J. E. , M'Gonigle, L. K. , & Resh, V. H. (2014). Changes in occurrence, richness, and biological traits of dragonflies and damselflies (Odonata) in California and Nevada over the past century. Biodiversity and Conservation, 23(8), 2107–2126. 10.1007/s10531-014-0707-5

[ece38648-bib-0005] Blackburn, T. , Gaston, K. , Greenwood, J. , & Gregory, R. (1998). The anatomy of the interspecific abundance‐range size relationship for the British avifauna: II. Temporal Dynamics. Ecology Letters, 1(1), 47–55. 10.1046/j.1461-0248.1998.00005.x

[ece38648-bib-0006] Bried, J. T. , Dillon, A. M. , Hager, B. J. , Patten, M. A. , & Luttbeg, B. (2015). Criteria to infer local species residency in standardized adult dragonfly surveys. Freshwater Science, 34(3), 1105–1113. 10.1086/682668

[ece38648-bib-0007] Burnham, K. P. , Anderson, D. R. , & Huyvaert, K. P. (2011). AIC model selection and multimodel inference in behavioral ecology: some background, observations, and comparisons. Behavioral Ecology and Sociobiology, 65, 23–35. 10.1007/s00265-010-1029-6

[ece38648-bib-0008] Burnham, P. , & Anderson, D. R. (2000). Model selection and multimodel inference. A practical information‐theoretic approach. Springer.

[ece38648-bib-0009] Cardoso, P. , Barton, P. S. , Birkhofer, K. , Chichorro, F. , Deacon, C. , Fartmann, T. , Fukushima, C. S. , Gaigher, R. , Habel, J. C. , Hallmann, C. A. , Hill, M. J. , Hochkirch, A. , Kwak, M. L. , Mammola, S. , Noriega, J. A. , Orfinger, A. B. , Pedraza, F. , Pryke, J. S. , Roque, F. O. , … Samways, M. J. (2020). Scientists’ warning to humanity on insect extinctions. Biological Conservation, 242, 108426. 10.1016/j.biocon.2020.108426

[ece38648-bib-0010] Chichorro, F. , Juslen, A. , & Cardoso, P. (2019). A review of the relation between species traits and extinction risk. Biological Conservation, 237, 220–229. 10.1016/j.biocon.2019.07.001

[ece38648-bib-0011] Corbet, P. S. (1999). Dragonflies: Behaviour and Ecology of Odonata. Harley Books.

[ece38648-bib-0012] Corbet, P. S. , Suhling, F. , & Soendgerath, D. (2006). Voltinism of Odonata: A review. International Journal of Odonatology, 9, 1–44. 10.1080/13887890.2006.9748261

[ece38648-bib-0014] Dudgeon, D. , Arthington, A. H. , Gessner, M. O. , Kawabata, Z. , Knowler, D. J. , Leveque, C. , Naiman, R. J. , Prieur‐Richard, A. , Soto, D. , Stiassny, M. L. J. , & Sullivan, C. A. (2006). Freshwater biodiversity: importance, threats, status and conservation challenges. Biological Reviews, 81(2), 163–182. 10.1017/S1464793105006950 16336747

[ece38648-bib-0015] Freckleton, R. , Harvey, P. , & Pagel, M. (2002). Phylogenetic analysis and comparative data: A test and review of evidence. American Naturalist, 160(6), 712–726. 10.1086/343873 18707460

[ece38648-bib-0016] Fritz, S. A. , & Purvis, A. (2010). Selectivity in mammalian extinction risk and threat types: a new measure of phylogenetic signal strength in binary traits. Conservation Biology, 24(4), 1042–1051. 10.1111/j.1523-1739.2010.01455.x 20184650

[ece38648-bib-0017] Gaston, K. (2003). The structure and dynamics of geographical ranges. Oxford University Press.

[ece38648-bib-0018] Gilroy, J. J. , & Sutherland, W. J. (2007). Beyond ecological traps: perceptual errors and undervalued resources. Trends in Ecology and Evolution, 22(7), 351–356. 10.1016/j.tree.2007.03.014 17416438

[ece38648-bib-0019] Grewe, Y. , Hof, C. , Dehling, D. M. , Brandl, R. , & Brändle, M. (2013). Recent range shifts of European dragonflies provide support for an inverse relationship between habitat predictability and dispersal. Global Ecology and Biogeography, 22, 403–409. 10.1111/geb.12004

[ece38648-bib-0020] Hanski, I. (1999). Metapopulation Ecology. Oxford University Press.

[ece38648-bib-0021] Hanski, I. , & Ovaskainen, O. (2000). The metapopulation capacity of a fragmented landscape. Nature, 404(6779), 755–758. 10783887 10.1038/35008063

[ece38648-bib-0022] Harmon, L. J. , Weir, J. T. , Brock, C. D. , Glor, R. E. , & Challenger, W. (2008). GEIGER: investigating evolutionary radiations. Bioinformatics, 24(1), 129–131. 10.1093/bioinformatics/btm538 18006550

[ece38648-bib-0023] Ho, L. S. T. , & Ane, C. (2014). A Linear‐time algorithm for gaussian and non‐gaussian trait evolution models. Systematic Biology, 63(3), 397–408. 10.1093/sysbio/syu005 24500037

[ece38648-bib-0024] Hof, C. , Brändle, M. , & Brandl, R. (2006). Lentic odonates have larger and more northern ranges than lotic species. Journal of Biogeography, 33, 63–70. 10.1111/j.1365-2699.2005.01358.x

[ece38648-bib-0025] Hof, C. , Levinsky, I. , Araújo, M. B. , & Rahbek, C. (2011). Rethinking species’ ability to cope with rapid climate change. Global Change Biology, 17, 2987–2990. 10.1111/j.1365-2486.2011.02418.x

[ece38648-bib-0026] Ilvonen, J. J. , & Suhonen, J. (2016). Phylogeny affects host's weight, immune response and parasitism in damselflies and dragonflies. Royal Society Open Science, 3(11), 160421. 10.1098/rsos.160421 28018621 PMC5180119

[ece38648-bib-0027] Ives, A. R. , & Garland, T. Jr (2010). Phylogenetic logistic regression for binary dependent variables. Systematic Biology, 59(1), 9–26. 10.1093/sysbio/syp074 20525617

[ece38648-bib-0028] Jeppsson, T. , & Forslund, P. (2014). Species’ traits explain differences in Red list status and long‐term population trends in longhorn beetles. Animal Conservation, 17(4), 332–341. 10.1111/acv.12099

[ece38648-bib-0029] Kallimanis, A. S. , Kunin, W. E. , Halley, J. M. , & Sgardelis, S. P. (2005). Metapopulation extinction risk under spatially autocorrelated disturbance. Conservation Biology, 19, 534–546. 10.1111/j.1523-1739.2005.00418.x

[ece38648-bib-0030] Karjalainen, S. (2010). Suomen sudenkorennot. Kustannusosakeyhtiö Tammi.

[ece38648-bib-0031] Korkeamäki, E. , & Suhonen, J. (2002). Distribution and habitat specialization of species affect local extinction in dragonfly Odonata populations. Ecography, 25(4), 459–465. 10.1034/j.1600-0587.2002.250408.x

[ece38648-bib-0032] Kotiaho, J. , Kaitala, V. , Komonen, A. , & Päivinen, J. (2005). Predicting the risk of extinction from shared ecological characteristics. Proceedings of the National Academy of Sciences of the United States of America, 102(6), 1963–1967. 10.1073/pnas.0406718102 15671171 PMC548535

[ece38648-bib-0066] Kwak, M. L. , Heath, A. C. , & Cardoso, P. (2020). Methods for the assessment and conservation of threatened animal parasites. Biological Conservation, 248, 8696. https://www.sciencedirect.com/science/article/pii/S0006320720307540

[ece38648-bib-0033] Mattila, M. , Kotiaho, J. S. , Kaitala, V. , & Komonen, A. (2008). The use of ecological traits in extinction risk assessments: A case study on geometrid moths. Biological Conservation, 141(9), 2322–2328. 10.1016/j.biocon.2008.06.024

[ece38648-bib-0034] Mattila, N. , Kaitala, V. , Komonen, A. , Kotiaho, J. S. , & Päivinen, J. (2006). Ecological determinants of distribution decline and risk of extinction in moths. Conservation Biology, 20(4), 1161–1168. 10.1111/j.1523-1739.2006.00404.x 16922232

[ece38648-bib-0035] McCauley, S. J. , Davies, C. J. , Werner, E. E. , & Robeson, M. S. II (2014). Dispersal, niche breadth and population extinction: colonization rations predict range size in North American dragonflies. Journal of Animal Ecology, 83, 858–865. 10.1111/1365-2656.12181 24237364

[ece38648-bib-0036] Muenkemueller, T. , Lavergne, S. , Bzeznik, B. , Dray, S. , Jombart, T. , Schiffers, K. , & Thuiller, W. (2012). How to measure and test phylogenetic signal. Methods in Ecology and Evolution, 3(4), 743–756. 10.1111/j.2041-210X.2012.00196.x

[ece38648-bib-0037] Nylin, S. , & Bergstrom, A. (2009). Threat status in butterflies and its ecological correlates: how far can we generalize? Biodiversity and Conservation, 18(12), 3243–3267. 10.1007/s10531-009-9640-4

[ece38648-bib-0038] Orme, D. (2018). The caper package: comparative analyses of phylogenetics and evolution in R. https://CRAN.R‐project.org/package=caper

[ece38648-bib-0039] Outomuro, D. , & Johansson, F. (2019). Wing morphology and migration status, but not body size, habitat or Rapoport’s rule predict range size in North‐American dragonflies (Odonata: Libellulidae). Ecography, 42, 309–320. 10.1111/ecog.03757

[ece38648-bib-0040] Pagel, M. (1999). Inferring the historical patterns of biological evolution. Nature, 401(6756), 877–884. 10553904 10.1038/44766

[ece38648-bib-0041] Paradis, E. , Claude, J. , & Strimmer, K. (2004). APE: Analyses of phylogenetics and evolution in R language. Bioinformatics, 20(2), 289–290. 10.1093/bioinformatics/btg412 14734327

[ece38648-bib-0042] Patten, M. A. , Bried, J. T. , & Smith‐Patten, B. D. (2015). Survey data matter: predicted niche of adult vs breeding Odonata. Freshwater Science, 34(3), 1114–1122. 10.1086/682676

[ece38648-bib-0043] Pöyry, J. , Luoto, M. , Heikkinen, R. K. , Kuussaari, M. , & Saarinen, K. (2009). Species traits explain recent range shift of Finnish butterflies. Global Change Biology, 15, 732–743.

[ece38648-bib-0044] Pulliam, H. R. (1988). Sources, sinks, and population regulation. American Naturalist, 132(5), 652–661. 10.1086/284880

[ece38648-bib-0045] RCore Team (2018). A language and environment for statistical computing. R foundation for statistical computing.

[ece38648-bib-0046] Revell, L. J. (2012). phytools: an R package for phylogenetic comparative biology (and other things). Methods in Ecology and Evolution, 3(2), 217–223. 10.1111/j.2041-210X.2011.00169.x

[ece38648-bib-0047] Ricciardi, A. , & Rasmussen, J. B. (1999). Extinction rates of North American freshwater fauna. Conservation Biology, 13(5), 1220–1222. 10.1046/j.1523-1739.1999.98380.x

[ece38648-bib-0048] Richter, B. D. , Braun, D. P. , Mendelson, M. A. , & Master, L. L. (1997). Threats to imperiled freshwater fauna. Conservation Biology, 11(5), 1081–1093.

[ece38648-bib-0049] Rocha‐Ortega, M. , Rodriguez, P. , Bried, J. , Abbott, J. , & Cordoba‐Aguilar, A. (2020). Why do bugs perish? Range size and local vulnerability traits as surrogates of Odonata extinction risk. Proceedings of the Royal Society B: Biological Sciences, 287(1924), 20192645. 10.1098/rspb.2019.2645 PMC7209059 32228412

[ece38648-bib-0050] Rocha‐Ortega, M. , Rodriguez, P. , & Córdoba‐Aguilar, A. (2021). Geographical, temporal and taxonomic biases in insect GBIF data on biodiversity and extinction. Ecological Entomology, 46(4), 718–728.

[ece38648-bib-0051] Rundle, S. D. , Bilton, D. T. , Abbott, J. C. , & Foggo, A. (2007). Range size in North American *Enallagma* damseflies correlates with wing size. Freshwater Biology, 52, 471–477.

[ece38648-bib-0052] Seibold, S. , Gossner, M. M. , Simons, N. K. , Bluethgen, N. , Mueller, J. , Ambarli, D. , Ammer, C. , Bauhus, J. , Fischer, M. , Habel, J. C. , Linsenmair, K. E. , Nauss, T. , Penone, C. , Prati, D. , Schall, P. , Schulze, E. , Vogt, J. , Woellauer, S. , & Weisser, W. W. (2019). Arthropod decline in grasslands and forests is associated with landscape‐level drivers. Nature, 574(7780), 671–674. 10.1038/s41586-019-1684-3 31666721

[ece38648-bib-0053] Suarez‐Tovar, C. M. , Rocha‐Ortega, M. , Gonzalez‐Voyer, A. , Gonzalez‐Tokman, D. , & Cordoba‐Aguilar, A. (2019). The larger the damselfly, the more likely to be threatened: a sexual selection approach. Journal of Insect Conservation, 23(3), 535–545. 10.1007/s10841-019-00142-0

[ece38648-bib-0054] Suhling, F. , Sahlen, G. , Martens, A. , Marais, E. , & Schutte, C. (2006). Dragonfly assemblages in arid tropical environments: A case study from Western Namibia. Biodiversity and Conservation, 15(1), 311–332. 10.1007/s10531-005-2007-6

[ece38648-bib-0055] Suhonen, J. , Hilli‐Lukkarinen, M. , Korkeamäki, E. , Kuitunen, M. , Kullas, J. , Penttinen, J. , & Salmela, J. (2010). Local extinction of dragonfly and damselfly populations in low‐ and high‐quality habitat patches. Conservation Biology, 24(4), 1148–1153. 10.1111/j.1523-1739.2010.01504.x 20412087

[ece38648-bib-0056] Suhonen, J. , Korkeamäki, E. , Salmela, J. , & Kuitunen, M. (2014). Risk of local extinction of Odonata freshwater habitat generalists and specialists. Conservation Biology, 28(3), 783–789. 10.1111/cobi.12231 24405332

[ece38648-bib-0057] Swaegers, J. , Janssens, S. B. , Ferreira, S. , Watts, P. C. , Mergeay, J. , McPeek, M. A. , & Stocks, R. (2014). Ecological and evolutionary drivers of range size in *Coenagrion* damselflies. Journal of Evolutionary Biology, 27, 2386–2395. 25244423 10.1111/jeb.12481

[ece38648-bib-0058] Valle, K. J. (1952). Die Verbteitungsverhältnisse der ostfennoskandischen Odonaten (Zur Kenntnis der Odonatenfauna Finnlands 6.). Acta Entomologica Fennica, 10, 1–87.

[ece38648-bib-0059] Valtonen, P. (1980). Die Verbteirung der Finnischen Libellen (Odonata). Notulae Entomology, 60, 199–215.

[ece38648-bib-0060] van Klink, R. , Bowler, D. E. , Gongalsky, K. B. , Swengel, A. B. , Gentile, A. , & Chase, J. M. (2020). Meta‐analysis reveals declines in terrestrial but increases in freshwater insect abundances. Science, 368, 417–420. 10.1126/science.aax9931 32327596

[ece38648-bib-0061] van Swaay, C. (1990). An Assessment of the Changes in Butterfly Abundance in the Netherlands during the 20th‐Century. Biological Conservation, 52(4), 287–302. 10.1016/0006-3207(90)90073-X

[ece38648-bib-0062] Wagner, D. L. , Grames, E. M. , Forister, M. L. , Berenbaum, M. R. , & Stopak, D. (2021). Insect decline in the Anthropocene: Death by a thousand cuts. Proceedings of the National Academy of Sciences, 118(2), e2023989118. 10.1073/pnas.2023989118 PMC7812858 33431573

[ece38648-bib-0063] Waller, J. T. , & Svensson, E. I. (2017). Body size evolution in an old insect order: No evidence for Cope's Rule in spite of fitness benefits of large size. Evolution, 71(9), 2178–2193. 10.1111/evo.13302 28685868

[ece38648-bib-0064] Watkinson, A. R. , & Sutherland, W. J. (1995). Sources, Sinks and Pseudo‐Sinks. Journal of Animal Ecology, 64(1), 126–130. 10.2307/5833

[ece38648-bib-0065] Wickham, H. (2016). ggplot2: Elegant graphics for data analysis. Springer.

